# Does Semi-Rigid Instrumentation Using Both Flexion and Extension Dampening Spacers Truly Provide an Intermediate Level of Stabilization?

**DOI:** 10.1155/2013/738252

**Published:** 2013-04-11

**Authors:** Dilip Sengupta, Brandon Bucklen, Aditya Ingalhalikar, Aditya Muzumdar, Saif Khalil

**Affiliations:** ^1^Dartmouth-Hitchcock Medical Center, Orthopedics, One Medical Center Drive, Lebanon, NH 03756-0001, USA; ^2^Globus Medical Inc., Valley Forge Business Center, 2560 General Armistead Avenue, Audubon, PA 19403, USA

## Abstract

Conventional posterior dynamic stabilization devices demonstrated a tendency towards highly rigid stabilization approximating that of titanium rods in flexion. In extension, they excessively offload the index segment, making the device as the sole load-bearing structure, with concerns of device failure. The goal of this study was to compare the kinematics and intradiscal pressure of monosegmental stabilization utilizing a new device that incorporates both a flexion and extension dampening spacer to that of rigid internal fixation and a conventional posterior dynamic stabilization device. The hypothesis was the new device would minimize the overloading of adjacent levels compared to rigid and conventional devices which can only bend but not stretch. The biomechanics were compared following injury in a human cadaveric lumbosacral spine under simulated physiological loading conditions. The stabilization with the new posterior dynamic stabilization device significantly reduced motion uniformly in all loading directions, but less so than rigid fixation. The evaluation of adjacent level motion and pressure showed some benefit of the new device when compared to rigid fixation. Posterior dynamic stabilization designs which both bend and stretch showed improved kinematic and load-sharing properties when compared to rigid fixation and when indirectly compared to existing conventional devices without a bumper.

## 1. Introduction

Fusion using rigid pedicle screw-rod instrumentation is a conventional surgical treatment for mechanical back pain due to disc degeneration when nonoperative treatment has failed. In spite of this standard, it is associated with implant-related failures such as screw breakage or loosening. Screw breakage or loosening have been reported in the literature to range from 1% to 11.2% of the screws inserted [1–7]. It has been shown to be affected by a number of factors such as screw design, the number of levels fused, anterior column load-sharing, bone density, the presence of pseudoarthrosis, and its use in burst fractures [3, 4, 8–10]. While in multilevel fusion, bone density and burst fracture applications are more related to patient pathology and indications; all other factors are more dependent on implant design and biomechanics. Anterior column load-sharing is negatively affected by the absence of interbody support and higher stiffness of posterior fixation devices [3, 11]. Adjacent segment degeneration (ASD) has also been recognized as a potential long-term complication of rigidly instrumented fusion [12–17]. While there is some debate surrounding the causality of the disease (whether it is mechanical factors or a natural degenerative progression), a review of 271 articles found a higher rate of symptomatic ASD in 12%–18% of patients fused with rigid transpedicular instrumentation. In spite of these disadvantages, it is proven that implant rigidity is required to achieve successful fusion.

The challenge for surgeons, biomechanists, and engineers has been to determine and develop an optimally stiff device that will provide enough rigidity across a destabilized spinal segment while simultaneously sharing load with the fusion mass. Posterior fixation devices have evolved from larger diameters and stiffer materials (6.5 mm cobalt chromium/stainless steel) to smaller diameters and less stiff or semi-rigid materials (5.5 mm poly ether ether ketone (PEEK)), respectively. Semi-rigid fixation or dynamic stabilization devices such as PEEK rods, titanium rods with helical grooves, and polymeric spacers with an interwoven cord tethered between pedicle screws have been designed to increase load-sharing in an attempt to induce compression on the bone graft and accentuate the concept of bone remodeling as first credited by Wolff [18]. Examples of such devices are Isobar TTL (Scient'x, Maitland, FL), a metal rod with disc springs, the CD Horizon Legacy PEEK rod (Medtronic Sofamor Danek, Memphis, TN), and Dynesys Dynamic Stabilization System (Zimmer, Warsaw, IN) consisting of a polymeric dampener and posterior tensioning cord. Semi-rigid fixation devices attempt to offload adjacent levels, but most studies show the stiffness of these constructs to be too high to have much of an effect on adjacent level loading [18–21]. These devices have also been clinically recommended for stabilization and modulation of the load distribution across mildly degenerated discs in an attempt to alleviate discogenic back pain and potentially enable regeneration of disc cells [22, 23].

In this particular study, the TRANSITION Stabilization System (Globus Medical, Inc., Audubon, PA) was utilized as the method of semi-rigid stabilization. The device was designed to bend and stretch by incorporating two polymeric spacers: one strategically placed above the cranial pedicle screw and the other between the pedicle screws, to allow a resistance to flexion, and a natural compression across the joint, respectively. We hypothesize that the compressibility across the surgical level may have implications on both the index and adjacent levels, but to what degree remains unknown.

The aim of this study was to evaluate the implanted and adjacent level kinematics and load-sharing effects of the human lumbosacral spine implanted with a semi-rigid fixation device, TRANSITION, compared to rigid fixation, and the historical performance of conventional semi-rigid devices. In this study, the injury model of the motion segment was created by a decompression involving facetectomy. 

## 2. Materials and Methods

### 2.1. Specimen Preparation

**Figure 1:**
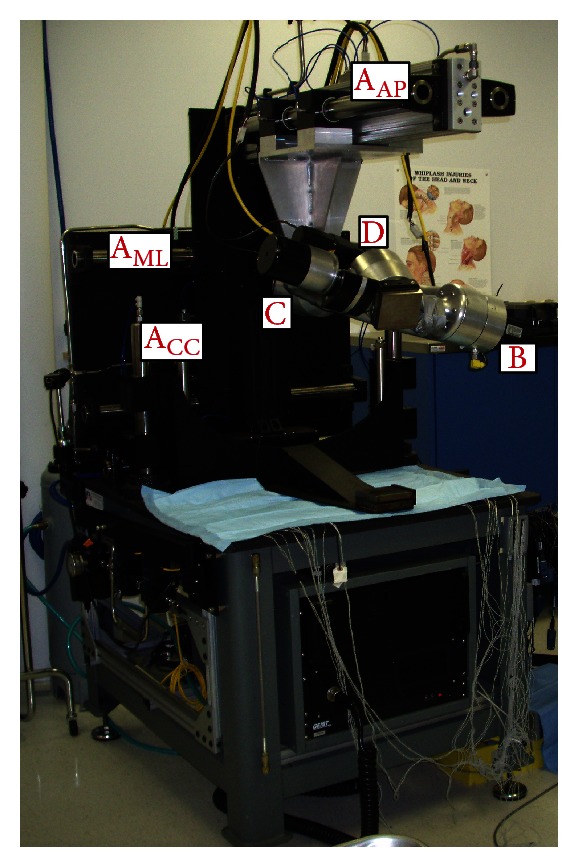
Six degree-of-freedom testing apparatus, allowing unconstrained motion and rotations. Three motors, each placed in a physiological rotation direction providing pure rotations, while translational guide rails allow the forces to redistribute according to the kinematic properties of the spine. A_AP_: guide rail with air bearings (anterior-posterior), A_ML_: guide rail with air bearings (medial-lateral), A_CC_: guide rail with air bearings (cephalad-caudal), B: flexion-extension motor, C: lateral bending motor, and D: axial rotation motor.

All spines were radiographed to ensure the absence of fractures, deformities, and any metastatic disease. The spines were stripped of paravertebral musculature while preserving the spinal ligaments, joints, and disk spaces. Subsequently, they were mounted at L1 rostrally and S1 caudally in a three-to-one mixture of Bond Auto Body Filler and fiberglass resin (Bondo MarHyde Corp., Atlanta, GA). The spine was then affixed to a six degree-of-freedom (6-DOF) testing apparatus, and pure unconstrained bending moments were applied in the physiological planes of the spine at room temperature using a multidirectional hybrid flexibility protocol [24]. The 6-DOF machine applied unconstrained loading through the application of three cephalad stepper motors placed in each of the three physiological rotation axes (Figure 1). Moreover, the supports were mounted on air bearings to provide near frictionless resistance to the natural kinematics of the spine. Plexiglas markers, each having three infrared light-emitting diodes, were secured rigidly to each vertebral body via bone screws to track its motion with Optotrak Certus (NDI, Inc. Waterloo, Canada) motion analysis system. The location of the markers (denoting a rigid body) was approximately aligned sagitally along the curvature of the spine. The Optotrak Certus software was able to superimpose the coordinate systems of two adjacent vertebral bodies in order to inferentially determine the relative eulerian rotations in each of the three planes.

### 2.2. Device Descriptions

**Figure 2:**
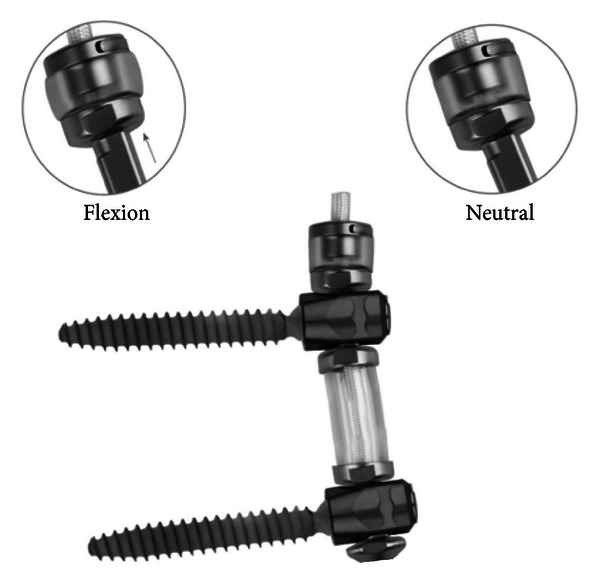
The TRANSITION Stabilization System. The cephalad bumper shown in neutral and flexed position.

The semi-rigid device which can both bend and stretch (TRANSITION) is composed of titanium, polycarbonate urethane (PCU), and polyethylene terephthalate (PET) (Figure 2). Essentially, instead of a rod, a PCU spacer is placed between the pedicle screws, while a central PET cord, which runs from top to bottom, provides resistance to stretching (namely flexion). The cord is not tethered to the screws, like conventional devices, but is passed through spools which are the attachment point of the pedicle screws. The spools are 5.5 mm thick at the portion which fits into the pedicle screw. Above the cranial pedicle screw is another PCU spacer which is compressed when the cord is in tension (flexion). The rigid rods tested were standard 5.5 mm diameter titanium rods (REVERE Stabilization System, Globus Medical). Both devices were locked in place through the same screws, having the same tulip, and same locking caps. Comparisons to historical controls or so-called conventional dynamic stabilization devices are primarily focused on Dynesys Dynamic Stabilization System (Zimmer, Warsaw, IN) but could also include Isobar TTL (Scient'x, Maitland, FL), CD Horizon Legacy PEEK rod (Medtronic Sofamor Danek, Memphis, TN), or others. Dynesys has been by far the most extensively studied, biomechanically and clinically.

### 2.3. Test Groups

**Figure 3:**
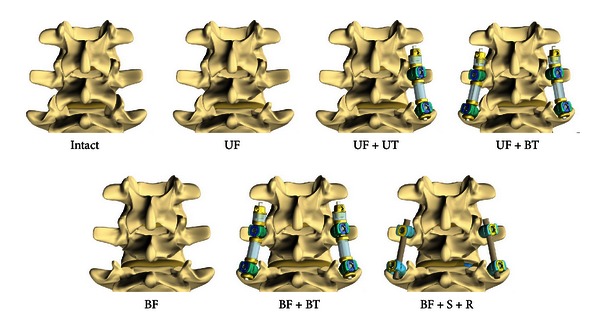
Surgical testing sequence. (1) Intact; (2) unilateral facetectomy (UF); (3) UF and unilateral TRANSITION device (UF + UT); (4) UF and bilateral TRANSITION device (UF + BT); (5) bilateral facetectomy (BF); (6) BF and bilateral TRANSITION device (BF + BT); (7) BF and bilateral rigid fixation with interbody spacer (BF + S + R).

Nine intact fresh human cadaver lumbosacral spines (L1-S1) were tested by applying a pure moment of ±8 Nm, according to the test standards for lumbar spine [25]. The specimens consisted of 6 males and 3 females, with an average age of 53 ± 10 years. The hybrid protocol for testing adjacent level effects was applied, as described by Panjabi [24]. Initially, the total L1-S1 range of motion (ROM) was determined in an individual intact specimen. In all subsequent tests for the respective specimen, the displacement of the spine was ranged to the intact total ROM values in flexion (F), extension (E), lateral bending (LB), and axial rotation (AR). A series of three load/unload cycles were performed for each motion with data analysis based on the final cycle. The first five specimens were tested for unilateral facetectomy and unilateral stabilization of L4-L5 segment in the following sequence (Figure 3): (1) intact; (2) unilateral facetectomy (UF); (3) UF and unilateral TRANSITION PDS device (UF + UT); and (4) UF and bilateral TRANSITION PDS device (UF + BT). All the nine specimens (including the previous five unilateral models) were tested for bilateral facetectomy and bilateral stabilization at the L4-L5 segment in the following sequence: (1) intact; (5) bilateral facetectomy (BF); (6) BF and bilateral TRANSITION PDS device (BF + BT); and (7) BF and bilateral rigid fixation (pedicle screws and titanium rod, REVERE Stabilization System, Globus Medical) with interbody spacer (Sustain-O, Globus Medical) (BF + S + R). The numbers in parenthesis indicate the construct number identifying the test condition, in the rest of this paper. Disc pressure was measured using miniature pressure transducers (width = 1.5 mm; height = 0.3 mm, Precision Measurement Co., Ann Arbor, MI) inserted at the adjacent levels, in the posterior half of the disc space, confirmed by sagittal radiographs [26]. The transducers were configured using C-DAQ (National Instruments, Austin, TX) data acquisition module.

### 2.4. Data Interpretation

Several comparisons were made to evaluate any statistical differences between constructs 1 and 7. The unilateral model (constructs 1, 2, 3, and 4) was evaluated separately from the bilateral model (constructs 1, 5, 6, and 7). Statistical comparisons were completed using a single factor, repeated measures analysis of variance (ANOVA). In all cases to alleviate inhomogeneity of variance, log transforms in the form of log_10_ (rawdata + 1) were applied to the raw data. Comparisons were made with a probability of type I error, *α* = 0.05, using Tukey's *post hoc *comparison for equal sample size (*n* = 5 unilateral and *n* = 9 bilateral). Intradiscal pressure (IDP) profiles were normalized according to the neutral zone “base pressure” such that the only changes between the base pressure and the pressure at maximum displacement were recorded according to Schmoelz et al. [13]. When the percentage change is discussed, unless otherwise stated, the percentages are calculated through differences in normalized ROM of surgical groups, when normalized to the intact spine motion (100%).

## 3. Results

### 3.1. Unilateral Model

**Figure 4:**
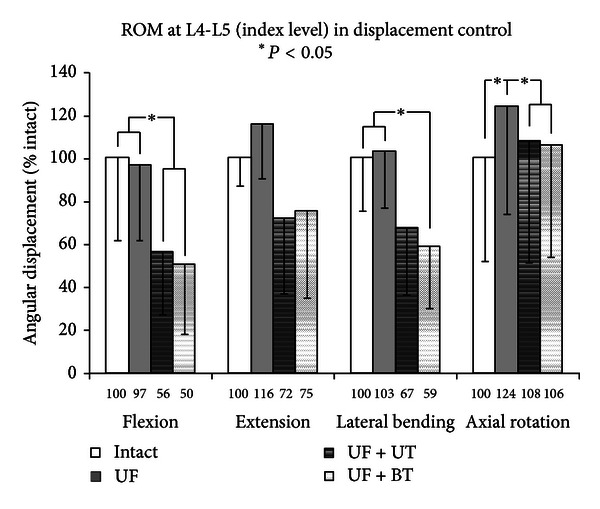
Index surgical level results of multidirectional flexibility testing for constructs 1, 2, 3, and 4 (unilateral model).

The range of motion (ROM) was determined for each surgical construct of the unilateral injury model (Figure 4), and *post hoc *comparisons were tabulated. Unilateral facetectomy (UF) did not cause any significant destabilization in flexion, extension, or lateral bending but increased rotation significantly (124% of intact; *Q* > *Q*_.05_, 7.9 > 4.2). The stabilization of the unilateral injury with a unilateral TRANSITION (UF + UT) resulted in the reduction of motion which was significant in flexion and axial rotation (*F*: 58% of injury, *Q* > *Q*_.05_, 4.4 > 4.2; AR: 87% of injury, *Q* > *Q*_.05_, 5.7 > 4.2) but insignificant in extension (*E*: 62% of injury) and lateral bending (LB: 65% of injury). The stabilization of the unilateral injury with a bilateral TRANSITION (UF + BF) resulted in the reduction of motion which was significant in flexion, lateral bending, and axial rotation (*F*: 52% of injury, *Q* > *Q*_.05_, 5.4 > 4.2; LB: 57% of injury, *Q* > *Q*_.05_, 5.1 > 4.2; AR: 85% of injury, *Q* > *Q*_.05_, 6.0 > 4.2) but insignificant in extension (*E*: 65% of injury). With respect to intact, the stabilization with a unilateral TRANSITION (UF + UT) resulted in the reduction of motion which was significant in flexion (*F*: 56% of intact, *Q* > *Q*_.05_, 4.6 > 4.2) but insignificant in extension (*E*: 72% of intact), lateral bending (LB: 67% of intact), and axial rotation (AR: 108% of intact). With respect to intact, stabilization with a bilateral TRANSITION (UF + BF) resulted in reduction of motion which was significant in flexion and lateral bending (*F*: 50% of intact, *Q* > *Q*_.05_, 5.6 > 4.2; LB: 59% of intact, *Q* > *Q*_.05_), but insignificant in extension (*E*: 75% of intact) and axial rotation (AR: 106% of intact). 

Increased motion due to the UF injury was expected to lead to reduced motions at the immediate adjacent levels in a displacement control protocol (Table 1). This was generally true (especially for L3-L4), but the reduced motions were small and insignificant, except in axial rotation. The stabilization with the PDS system reduced ROM at L4-L5, and, as expected, produced larger ROM at the adjacent levels, which reached significance (with respect to injury) only in lateral bending (L3-L4: UF + UT, 107% of injured, *Q* > *Q*_.05_, 4.7 > 4.2; L3-L4: UF + BT, 108% of injured, *Q* > *Q*_.05_, 5.3 > 4.2; L5-S1: UF + UT, 110% of injured *Q* > *Q*_.05_, 4.5 > 4.2; L5-S1: UF + BT, 112% of injured, *Q* > *Q*_.05_, 5.2 > 4.2) and axial rotation (L3-L4: UF + UT, 104% of injured, *Q* > *Q*_.05_, 4.6 > 4.2; L3-L4: UF + BT, 106% of injured, *Q* > *Q*_.05_, 5.6 > 4.2). There were few differences between unilateral stabilization (UF + UT) and bilateral stabilization (UF + BT) on adjacent level motion.

With respect to intact, adjacent level motion was significantly increased in lateral bending at L5-S1 by both PDS constructs (UF + UT: 114% of intact, *Q* > *Q*_.05_, 6.4 > 4.2; UF + BT: 116% of intact, *Q* > *Q*_.05_, 7.1 > 4.2).

Intradiscal pressure measurements of adjacent levels (Table 1) showed greater differences between intact and injury groups than what was seen kinematically. Therefore, even small changes in kinematics may translate to large changes in load-sharing properties. Statistically, in lateral bending, unilateral injury stabilized with a bilateral TRANSITION (UF + BT) was the only construct to produce significantly more adjacent level pressure than the corresponding level of the unilaterally injured spine (L3-L4: 131% of injured, *Q* > *Q*_.05_, 7.5 > 4.2) and the intact spine (L3-L4: 127% of intact, *Q* > *Q*_.05_, 5.7 > 4.2). With respect to the intact spine, both unilateral TRANSITION (UF + UT) and bilateral TRANSITION (UF + BT) produce significantly more adjacent level pressure in flexion (L5-S1: UF + UT, 161% of intact, *Q* > *Q*_.05_, 4.7 > 4.2; L3-L4: UF + BT, 220% of intact, *Q* > *Q*_.05_, 5.7 > 4.2; L5-S1: UF + BT, 207% of intact, *Q* > *Q*_.05_, 6.7 > 4.2).

### 3.2. Bilateral Model

**Figure 5:**
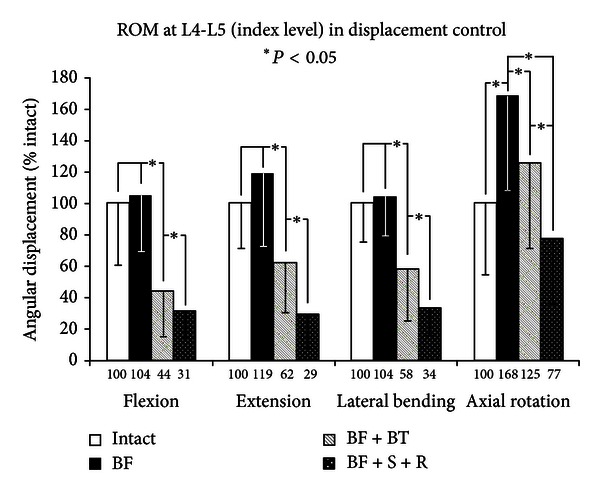
Index surgical level results of multidirectional flexibility testing for constructs 1, 4, 5, and 6 (bilateral model).

The range of motion (ROM) was determined for each surgical construct of the bilateral injury model (Figure 5), and *post hoc *comparisons were tabulated. Destabilization after BF increased the ROM in all directions, but this reached statistical significance only in axial rotation (AR: 168% of intact, *Q* > *Q*_.05_, 8.0 > 3.9). Again, in flexion and lateral bending, similar statistical trends were seen, revealing that BF + BT provided significant stabilization with respect to intact (F: 44% of intact, *Q* > *Q*_.05_, 15.1 > 3.9; LB: 58% of intact, *Q* > *Q*_.05_, 7.8 > 3.9) and BF (F: 42% of injury, *Q* > *Q*_.05_, 16.2 > 3.9; LB: 56% of injury, *Q* > *Q*_.05_, 8.3 > 3.9). In extension, the bilateral injury produced larger motions (119%) when compared to intact. 

The trend of index level motion follows the model BF + S + R < BF + BT < BF, where all constructs were statistically different than one another. The stabilization with TRANSITION PDS device reduced the ROM values, which were, in terms of intact, 44% (*Q* > *Q*_.05_, 15.1 > 3.9), 62% (*Q* > *Q*_.05_, 4.2 > 3.9), 58% (*Q* > *Q*_.05_, 7.8 > 3.9), and 125% (*Q* < *Q*_.05_, 3.3 < 3.9), while rigid fixation resulted in ROM values of 31% (*Q* > *Q*_.05_, 19.5 > 3.9), 29% (*Q* > *Q*_.05_, 8.7 > 3.9), 34% (*Q* > *Q*_.05_, 13.6 > 3.9), and 77% (*Q* < *Q*_.05_, 3.8 < 3.9) in *F*, *E*, LB, AR, respectively. Compared to the BF, and stabilization with TRANSITION PDS device reduced the ROM values, which were, in terms of injury, 42% (*Q* > *Q*_.05_, 16.2 > 3.9), 52% (*Q* > *Q*_.05_, 5.5 > 3.9), 56% (*Q* > *Q*_.05_, 8.3 > 3.9), and 74% (*Q* > *Q*_.05_, 4.7 > 3.9), while rigid fixation resulted in ROM values of 30% (*Q* > *Q*_.05_, 20.6 > 3.9), 24% (*Q* > *Q*_.05_, 10.0 > 3.9), 33% (*Q* > *Q*_.05_, 14.1 > 3.9), and 46% (*Q* > *Q*_.05_, 11.9 > 3.9) in *F*, *E*, LB, and AR, respectively.

**Figure 6:**
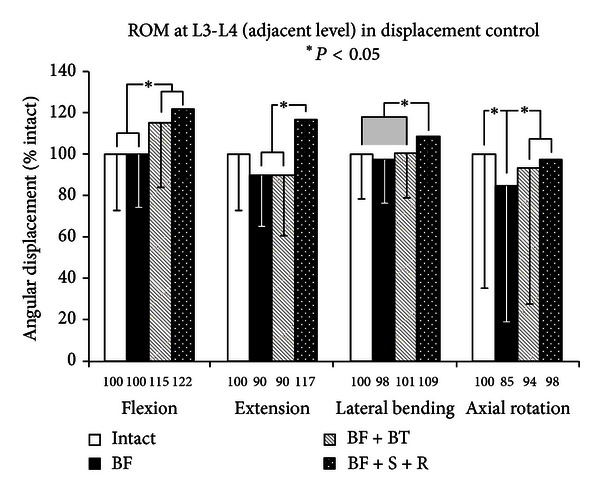
Cranial adjacent level results of multidirectional flexibility testing for constructs 1, 5, 6, and 7.

**Figure 7:**
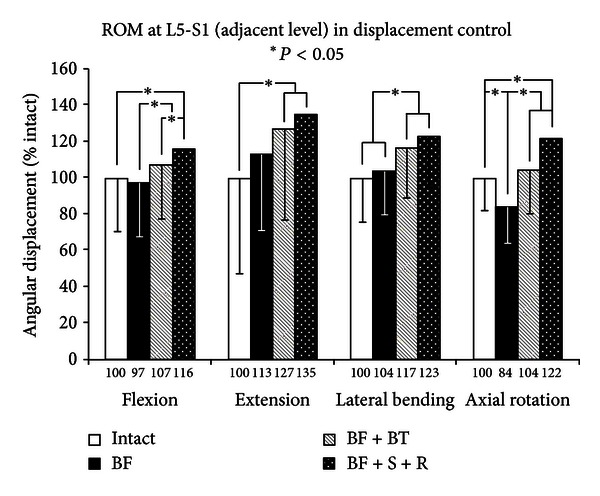
Caudal adjacent level results of multidirectional flexibility testing for constructs 1, 5, 6, and 7.

Increased motion due to the BF injury at the index level is expected to lead to reduced motions at the immediate adjacent levels in a displacement control protocol (Figures 6 and 7). This was generally correct, but the reduced motions were small and insignificant, except for axial rotation where BF was significantly less than intact (*P* < 0.05) (except for L5-S1). The stabilization at L4-L5 increased the ROM at both the adjacent levels and the trend followed the model BF + S + R ≥ BF + BT ≥ BF for all loading modes at both L3-L4 and L5-S1, indicating the utility of semi-rigid stabilization to offset adjacent level effects caused by rigid instrumentation. Nevertheless, this trend was not always large enough to warrant significance. 

**Figure 8:**
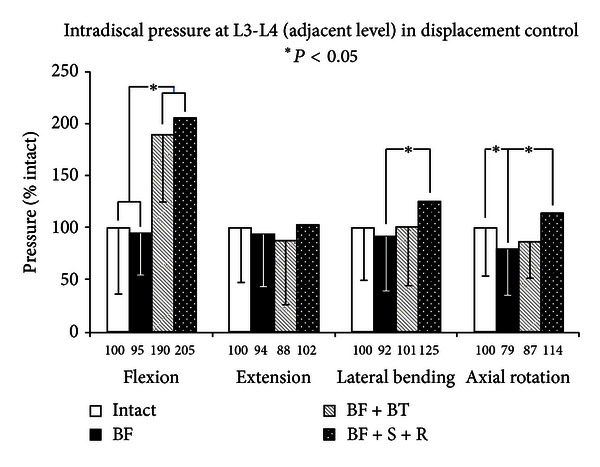
Cranial adjacent level intradiscal pressures of multidirectional flexibility testing for constructs 1, 5, 6, and 7.

**Figure 9:**
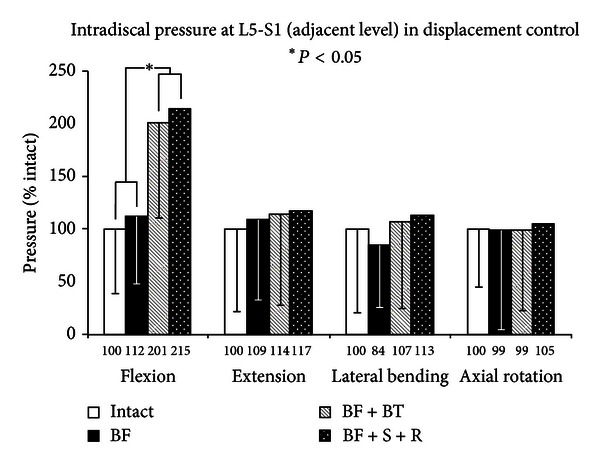
Caudal adjacent level intradiscal pressures of multidirectional flexibility testing for constructs 1, 5, 6, and 7.

The load-bearing effect at the adjacent levels, as measured by intradiscal pressure, (Figures 8 and 9) demonstrated very similar trends to ROM, that is, the IDP was decreased or unchanged after facetectomy at the L4-L5 level and increased with PDS stabilization, with an even greater increase with rigid stabilization. The increase in adjacent segment pressure after rigid stabilization was more pronounced at the cranial (L3-L4) level than the caudal (L5-S1) level, reaching a significant level, with respect to injury in flexion (209% of injured, *Q* > *Q*_.05_, 7.6 > 3.9), lateral bending (136% of injured, *Q* > *Q*_.05_, 5.5 > 3.9), and axial rotation (144% of injured, *Q* > *Q*_.05_, 5.4 > 3.9) at L3-L4, but only in flexion (192% of injured, *Q* > *Q*_.05_, 7.2 > 3.9) at L5-S1. While adjacent segment ROM changes were more pronounced in rotation, the increase in adjacent segment pressure was most noticeable in flexion. At the cranial adjacent level (L3-L4), while the ROM in flexion was increased to 122% after rigid fixation, the corresponding disc pressure was increased to 205% of the intact value. The stabilization with PDS also significantly increased the adjacent segment pressures in flexion, but the increase was smaller (190%) than with rigid fixation (*P* < 0.05). Therefore, though a strong relationship exists between ROM and IDP changes at the adjacent segments, it shows a nonlinear phenomenon in flexion. Additionally, though the use of the particular PDS device reduced the adjacent level pressure, it did not restore it near the intact value in flexion. Whether this would translate into potential alleviation of adjacent level stresses needs to be corroborated with clinical evidence. The remaining ROM and IDP trends are very similar, though higher variation (standard deviations) in the measurement of pressure resulted in very little significance and no significance between BF + BT and BF + S + R in any loading mode. 

## 4. Discussion

Conventional rigid fusion in the surgical treatment for chronic low back pain has some negative side effects such as the potential for adjacent segment degeneration and screw loosening. The concept of semi-rigid or dynamic stabilization has evolved to possibly prevent such degeneration, if it is not a function of natural disease progression, mainly through the reduction of stress at the adjacent segments. Soft-stabilization devices were developed to permit load-sharing with the anterior column to accomplish solid fusion and, at the same time, provide a softer posterior implant stiffness. Consequently, semi-rigid instrumentation is expected to lower screw breakage associated with transmission of forces through posterior instrumentation as opposed to through the anterior column. While there is some disparity between the potential uses of PDS systems (whether they are for reducing adjacent level degeneration or for promoting fusion through load-sharing), the ubiquitousness of such systems cannot be ignored. Their prevalence currently has more to do with dissatisfaction with conventional fusion than a proven efficacy. This study attempts to characterize the biomechanical efficacy of a select system. The clinical efficacy has yet to be determined. It remains to be seen if “soft fusion” can be achieved and if, in the presence of boney ingrowth with weaker mechanical properties, adjacent level effects can be ameliorated. 

The purpose of this study was to evaluate the stability of using a posterior dynamic stabilization (PDS) device which differs from conventional PDS devices in two ways: (1) by the addition of both flexion and extension dampening materials; and (2) by the addition of titanium spools (attached to the screw heads) which slide along the PET cord. The primary aim was to compare this device to rigid fixation with pedicle screws and rods. The hypothesis is that the new PDS design will load-share with the surgical level more effectively, therefore minimizing the over-load effect of the adjacent levels compared to the conventional rigid and PDS devices.

Both the PDS and rigid devices produced significant stabilization, but a consistent and significant trend of increased flexibility was observed in all loading modes for BF + BT (TRANSITION) when compared to BF + S + R (rigid). TRANSITION led to ROM values which were, in terms of intact, 44%, 62%, 58%, and 125% in *F*, *E*, LB, and AR, respectively, while rigid fixation resulted in ROM values of 31%, 29%, 34%, and 77%. Gédet et al. reported (load control protocol using a follower load and partial injury including a 25% nucleotomy) that Dynesys system provided stabilization when compared to intact values of ~20%, 40%, 40%, and 100% for *F*, *E*, LB, and AR, respectively [27]. The data from the current study showed a higher ROM baseline because of the facetectomy as opposed to nucleotomy as the injury model but the stabilization effect followed a similar pattern. A separate study, investigating Dynesys in a more severe injury model without axial preload, revealed that PDS restored motion to ~20%, 100%, 27%, and 130% of the intact values [14]. While it is difficult to directly compare the magnitudes reported in the literature sources to the current data, due to differences in test protocols, injury models, and the use of follower loads, the pattern in data is still comparable. 

The PDS device used in this study resulted in kinematic and load-sharing trends which appear different when compared to trends observed in conventional PDS designs within the literature [13, 14]. The majority of data in the literature show Dynesys behaves more rigid in flexion, almost comparable to rigid fixation, and less rigid in extension. On the contrary, the data from the present study show a more uniform rigidity in ROM across flexion, extension, and lateral bending. This inference is only based on indirect comparisons. In terms of load-sharing effect, the literature showed Dynesys responds to extension by total load-bearing of the implant, resulting in negative pressure in the disc at the index level [13]. This study cannot comment on load-sharing at the index level because the rigid rod construct was tested with an interbody spacer, precluding the simultaneous use of a pressure transducer. Comparisons of this construct with the PDS construct would not have been possible; therefore, both were excluded. Nevertheless, the adjacent level effects consistently reveal that the hypermobility of rigid fixation was reduced via TRANSITION. Moreover, the amount of reduction was uniform across the loading modes, not favoring extension over flexion. In rotation, more motion was allowed and not limited through the bumper mechanism. Yet, rotation itself is much less of a problem in a degenerated lumbar spine and is infrequently diagnosed as a cause of pain.

In a finite element study by Schmidt et al., the authors predicted the performance of PDS devices in different loading modes, as a function of polymer properties [28]. The material properties of posterior instrumentation were input in the analysis in terms of the bending stiffness and axial stiffness, axial stiffness referring to purely compressing the polymer spacer and bending stiffness similar to folding the spacer. The difference in bending stiffness between a PCU spacer and rigid rod is expected to be larger than their difference in axial stiffness. In that study, the authors concluded that, in each loading mode, the resulting ROM of an L4-L5 segment with posterior instrumentation involved a combination of both bending and axial stiffness. However, in flexion-extension, the relationship was mostly determined through axial stiffness, while in lateral bending and axial rotation, both stiffness parameters played a role. Extrapolating these results to PDS findings helps explain the relative rigidity of PDS devices in flexion-extension, which, despite a polymer spacer, are significantly stabilized with respect to intact values. Moreover, their findings predict that materials with high bending flexibility, such as PCU, would respond with increased motion in lateral bending and axial rotation. These conclusions are consistent with the results reported here as well as other studies. In this study, the extra polymeric material added through the spacer and bumper can be expected to add to the overall flexibility of conventional PDS devices, especially in lateral bending and axial rotation.

The PDS test device reduced adjacent level hypermobility caused by rigid fixation. The trend of adjacent level motions followed the model BF + S + R ≥ BF + BT ≥ BF for all loading modes at both L3-L4 and L5-S1, indicating the utility of semi-rigid stabilization to offset adjacent level effects. While this trend is encouraging to alleviate adjacent level stresses, its clinical relevance needs to be proven. The question “How much is off-loading ideal?” remains to be answered. Nevertheless, the new PDS device produced significantly smaller motions than rigid fixation at the adjacent levels, in flexion (only at L5-S1), extension (only at L3-L4), and lateral bending (only at L3-L4). 

Intradiscal pressure measurements at the adjacent level reflected the same trends as the ROM, but, in flexion, the relationship between ROM and IDP was nonlinear. For example, a 22% increase in L3-L4 level motion caused by L4-L5 rigid fixation, resulted in 105% increase in the IDP value. Moreover, the stabilization with PDS device (BF + BT) was not able to restore these large pressure that increases to near the intact value. If adjacent level disease is indeed related to a physiological imbalance in load-sharing and kinematics of segments juxtaposed to the fusion site, then the role of motion versus pressure on the rate of disease progression needs to be determined. Since these factors are nonlinearly related, restricting the motion may not be sufficient at buffering the load-sharing effects on the adjacent level.

There were certain limitations in this study. One objective was to relate the biomechanical differences observed between this study and those found on the widely studied conventional device, Dynesys. The ideal way to evaluate the difference was to compare TRANSITION versus Dynesys directly. In the current study, this comparison was indirect from the literature data. The reason behind this was that testing TRANSITION and Dynesys on the same specimen was not possible because the pedicle screws are different in the two systems, and the reinsertion of the pedicle screws in the same specimens introduces unacceptable errors because of loosening at the screw-bone interface. Removing the bumper alone from the TRANSITION does not make it comparable to Dynesys. The second limitation of this study was the bilateral facetectomy injury model, which may not be the most common scenario of a decompression clinically. However, facetectomy produced considerable instability, possibly more than what can be achieved by nucleotomy alone. The injury model was chosen because of the benefit of having a greater degree of instability (or worst-case scenario). Thirdly, testing pedicle screws and rods without an interbody device would have provided some information in the comparison of rigid rods and TRANSITION. Nevertheless, the authors were predominately interested in seeing the maximum change in the rigidity between interbody fusion with internal fixation and semi-rigid posterolateral fusion. Lastly, there is a certain amount of error introduced via suboptimal device placement which can occur via difficulty in the anatomy, irregular curvatures, or even screw placement. The PDS device considered made use of individually sized PCU spacers which were trialed to appropriate length. The implants are also pre-assembled with a constant tension of 220 N, so there should never be a case where one side of the disc space is artificially tensioned more than the other. Therefore, device placement was not separately considered in the analysis of variance.

## 5. Conclusion

The semi-rigid fixation/dynamic stabilization device investigated in this study, which utilized posteriorly placed flexion and extension dampening materials, was able to reduce the motion (*P* < 0.05) at the surgical level in all modes, and the reduction in motion was significantly less in comparison to rigid internal fixation. The adjacent levels were off-loaded by the dynamic stabilization device, in terms of both motion and intradiscal pressure, though the effect was often insignificant. The new dynamic device provides more uniform reduction of motion at the surgical level in all directions, especially in flexion, as well as permits more uniform load-sharing when compared to conventional systems like Dynesys. The disc, which is a uniform load-bearing structure of homogeneous material properties, may, likewise, benefit from a device with uniform rigidity.

## Figures and Tables

**Table 1:** Unilateral model (construct 1, 2, 3, and 4) adjacent level ROM and pressure. Brackets show which construct groups are significant.

	Intact	UF	UF + UT	UF + BT
	[1]	[2]	[3]	[4]
ROM (% of intact)				
Flexion				
L3-L4	Mean 100 (SD 23) [4]	Mean 101 (SD 20)	Mean 109 (SD 24)	Mean 111 (SD 27) [1]
L5-S1	Mean 100 (SD 32)	Mean 98 (SD 26)	Mean 107 (SD 41)	Mean 108 (SD 35)
Extension				
L3-L4	Mean 100 (SD 11)	Mean 91 (SD 12)	Mean 101 (SD 16)	Mean 98 (SD 9)
L5-S1	Mean 100 (SD 26)	Mean 118 (SD 28)	Mean 133 (SD 43)	Mean 126 (SD 35)
Lateral bending				
L3-L4	Mean 101 (SD 16)	Mean 98 (SD16) [3, 4]	Mean 105 (SD 17) [2]	Mean 106 (SD 20) [2]
L5-S1	Mean 100 (SD 28) [3,4]	Mean 104 (SD 29) [3, 4]	Mean 114 (SD 31) [1, 2]	Mean 116 (SD 31) [1, 2]
Axial rotation				
L3-L4	Mean 100 (SD 31) [2, 3, 4]	Mean 89 (SD 29) [1, 3, 4]	Mean 93 (SD 28) [1, 2]	Mean 94 (SD 28) [1, 2]
L5-S1	Mean 100 (SD 22)	Mean 99 (SD 25)	Mean 110 (SD 27)	Mean 109 (SD 26)

Pressure (% of intact)				
Flexion				
L3-L4	Mean 100 (SD 42) [4]	Mean 144 (SD 33)	Mean 166 (SD 38)	Mean 220 (SD 76) [1]
L5-S1	Mean 100 (SD 58) [3, 4]	Mean 141 (SD 62)	Mean 161 (SD 53) [1]	Mean 207 (SD 82) [1]
Extension				
L3-L4	Mean 100 (SD 21)	Mean 74 (SD 26)	Mean 78 (SD 31)	Mean 99 (SD 24)
L5-S1	Mean 100 (SD 84)	Mean 103 (SD 78)	Mean 120 (SD 89)	Mean 113 (SD 96)
Lateral bending				
L3-L4	Mean 100 (SD 54) [4]	Mean 97 (SD 59) [4]	Mean 109 (SD 66) [4]	Mean 127 (SD 76) [1, 2, 3]
L5-S1	Mean 100 (SD 78)	Mean 90 (SD 70)	Mean 90 (SD 65)	Mean 94 (SD 70)
Axial rotation				
L3-L4	Mean 100 (SD 44)	Mean 92 (SD 33)	Mean 110 (SD 36)	Mean 81 (SD 21)
L5-S1	Mean 100 (SD 33)	Mean 85 (SD 35)	Mean 100 (SD 45)	Mean 87 (SD 33)
